# In Memoriam Sir Roy Yorke Calne December 30th, 1930 to January 6th, 2024

**DOI:** 10.3389/ti.2024.12692

**Published:** 2024-02-01

**Authors:** Neville Jamieson

**Affiliations:** Retired Transplant Surgeon, Cambridge, United Kingdom

**Keywords:** in memoriam Roy Yorke Calne, liver transplant, immunosuppression, art, tolerance

In the 1950s and early 1960s the hope of saving lives by carrying out life-saving organ transplants remained an impossible dream outside the special case of kidney transplants between identical twins. This had demonstrated that the technical surgical challenge could be overcome using techniques based on the work of Alexis Carrel at the beginning of the century but the challenges faced by the immune response had defied attempts to overcome them using radiation and the existing pharmacological options.

The challenge of developing solid organ transplantation from a dream to a reality fell to a number of surgeon scientists around the world who went on to be the founders and creators of our specialty. Of these pioneering figures, Roy Calne was a leading star throughout his life. After early unsuccessful experiments using irradiation in kidney transplants carried out at the Royal College of Surgeons Buxton Browne farm in the UK, he pursued an interest in novel chemical immunosuppressive agents triggered by the availability of 6 mercaptopurine and subsequently its oral analogue azathioprine in the laboratory of Joe Murray in Boston. This work was to be the foundation of the development of clinical transplantation into a life-saving reality based on chemical immunosuppression and marked the beginning of an era of clinical organ transplantation.

At this point in his career he was still a trainee but returned to the UK to become a consultant surgeon at the Westminster hospital with Professor Harold Ellis and subsequently moved to become Professor of Surgery at Cambridge University in 1965 at the age of 34—a testimony to the recognition of his early achievements. With his nephrology colleague Dr. David Evans he developed a haemodialysis programme in a dialysis unit close to his home where he also had an office and experimental laboratory, and established a viable clinical renal transplant programme at Addenbrookes Hospital. He followed the work of key transplant researchers around the world and developed a friendly rivalry with Tom Starzl in the United States, following Tom’s work with liver transplantation with keen interest and carrying out experimental liver transplants in the laboratory. Early observations of the apparent longer survival of porcine liver transplant recipients were to encourage his lifelong interest in the potential of inducing post transplant tolerance.

He was to go on to carry out the first orthotopic liver transplant in Europe in 1968. In the days before the acceptance of brain stem death testing this was from a DCD donor and because of a significant size disparity between donor and recipient also required the use of a novel surgical technique in the form of a “piggy back” caval reconstruction. Surgical innovation was to be a key component of his career and this was an early sign of his ability to adapt to complex surgical circumstances. He established a link with Roger Williams at Kings College Hospital in London which led to the establishment of the Kings/Cambridge joint liver transplant programme which was to continue for many years.

The outcomes of transplantation still offered room for improvement and he continued a keen interest in novel immunosuppressive agents alongside a career long interest in the possibility of achieving the holy grail of tolerance. He attracted scientists to work in his experimental laboratory and together with Dr. David White came across the novel agent Cyclosporin A (later Cyclosporine) which had been developed by Sandoz in Switzerland but was not felt to have any future practical use. They managed to persuade Sandoz this might not be the case and after discovering that the very insoluble agent could be dissolved in olive oil (the suggestion of a Greek visitor working in the laboratory at the time) went on to demonstrate its remarkable immunosuppressive properties allowing its first use in human transplantation in Cambridge in 1978. This led to a step change in kidney transplant outcomes and a renewed interest in transplantation of other organs.

Drug toxicity remained a challenge and novel agents and approaches continued as a major interest with key involvement in the development of Tacrolimus and Sirolimus and with the tempting hints of long term drug free tolerance suggested by experimental observations in murine liver graft recipients acting as a spur.

He maintained a lifelong practice in general surgery and later in his career was part of the first UK combined heart liver and heart lung liver transplant, the development of paediatric liver transplantation and intestinal transplantation. He came to wider public notice outside the transplant community with the transplant of an infant named Ben Hardwick who featured on “That’s Life” a popular UK TV programme hosted by Esther Rantzen—Ben was one of the early paediatric transplants in Cambridge in 1984.

Roy Calne was central in all of these events, becoming a leading figure in the newly forming national and international transplantation organisations and publishing more than a thousand papers in a wide range of medical journals together with numerous books on transplantation and general surgery. He was respected and admired by colleagues, locally, nationally and internationally. Over the years surgeons and scientists from around the world came to Cambridge to learn about all aspects of transplantation and work both clinically and in the laboratory before returning home to apply the skills they had learned in Cambridge. They were welcomed and made to feel at home and feel part of the extended family of the Cambridge Transplant Unit attending ward rounds, helping with transplants and attending social events at the Calne family home although these were not infrequently interrupted by the departure of teams (including the host and guests) to carry out transplants at the hospital.

Alongside his career in medicine he had many outside interests. He was a fellow of Trinity Hall in Cambridge and was an active and enthusiastic member of the College fellowship. Sports were important with squash and skiing in the winter and tennis in summer. There was an annual skiing trip jointly with Peter Morris and the Oxford transplant group which featured a Cambridge Oxford ski race featuring both clinical teams and family. A major feature of his life was painting in a variety of styles and themes and his work has featured in many exhibitions and many examples of his work can be found hanging in the corridors of Addenbrookes hospital where he spent his career in addition to featuring in national and international exhibitions. In later life he developed an interest in sculpture and produced a number of impressive bronze figures.

I worked with Roy for much of my career joining the transplant programme for the first time in 1979, an exciting time as this was the year after the first clinical use of Cyclosporine. I had the pleasure of working with him as a trainee and then as a colleague as transplantation developed with the Cambridge unit at its centre. Alongside our UK trainees I met a generation of young transplant clinicians and scientists attracted to Cambridge by Roy Calne’s reputation and drive who remain friends and who will remember the charismatic figure of Roy Calne with affection and admiration for his achievements.

His contributions were recognised nationally by the award of a knighthood in 1986, a Fellowship of the Royal Society in 1974, the “Pride of Britain” award in 2014 and internationally by multiple prizes and awards. He received the Lasker DeBakey prize jointly with Tom Starzl in 2012 in recognition of their joint contributions to the field of liver transplantation.

He is survived by his wife Patsy and six children and will remain in the memory of a generation of transplantation surgeons who had the pleasure of meeting him and working alongside him.


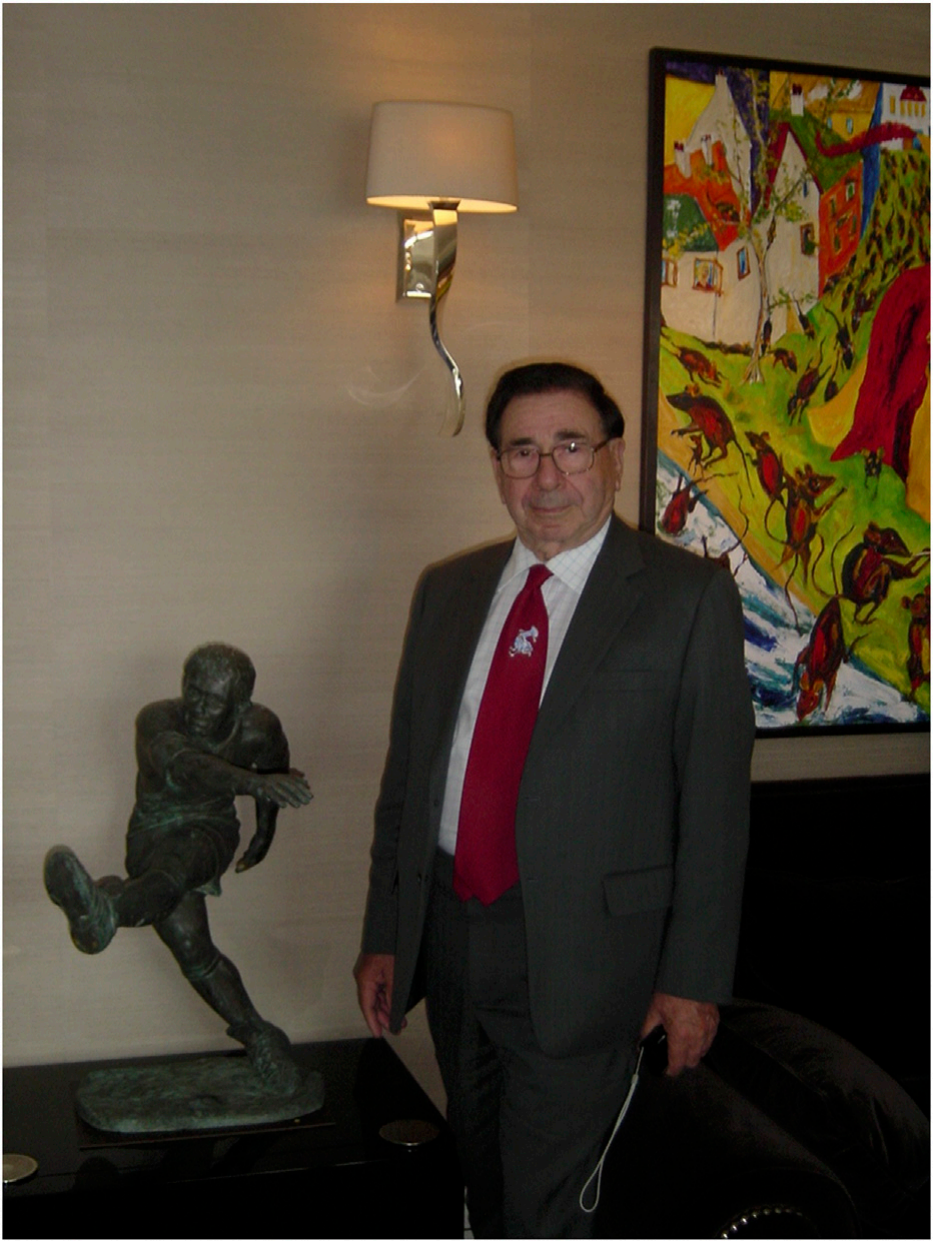


Sir Roy Calne, the artist with one of his bronze figures and one of his paintings.

## Data Availability

The original contributions presented in the study are included in the article/supplementary material, further inquiries can be directed to the corresponding author.

